# Detection of Gastrointestinal Pathogens from Stool Samples on Hemoccult Cards by Multiplex PCR

**DOI:** 10.1155/2017/3472537

**Published:** 2017-03-16

**Authors:** Martin Alberer, Nicklas Schlenker, Malkin Bauer, Kerstin Helfrich, Carolin Mengele, Thomas Löscher, Hans Dieter Nothdurft, Gisela Bretzel, Marcus Beissner

**Affiliations:** Department of Infectious Diseases and Tropical Medicine, Medical Centre, Ludwig-Maximilians-University (LMU), Munich, Germany

## Abstract

*Purpose*. Up to 30% of international travelers are affected by travelers' diarrhea (TD). Reliable data on the etiology of TD is lacking. Sufficient laboratory capacity at travel destinations is often unavailable and transporting conventional stool samples to the home country is inconvenient. We evaluated the use of Hemoccult cards for stool sampling combined with a multiplex PCR for the detection of model viral, bacterial, and protozoal TD pathogens.* Methods*. Following the creation of serial dilutions for each model pathogen, last positive dilution steps (LPDs) and thereof calculated last positive sample concentrations (LPCs) were compared between conventional stool samples and card samples. Furthermore, card samples were tested after a prolonged time interval simulating storage during a travel duration of up to 6 weeks.* Results*. The LPDs/LPCs were comparable to testing of conventional stool samples. After storage on Hemoccult cards, the recovery rate was 97.6% for* C. jejuni*, 100% for* E*.* histolytica*, 97.6% for norovirus GI, and 100% for GII. Detection of expected pathogens was possible at weekly intervals up to 42 days.* Conclusion*. Stool samples on Hemoccult cards stored at room temperature can be used in combination with a multiplex PCR as a reliable tool for testing of TD pathogens.

## 1. Introduction

The number of international travelers is continuously increasing and has passed the mark of one billion in 2013 for the first time. Travel destinations also include countries with low economic and hygienic standards; many of them are located in the tropical and subtropical regions. Therefore, travelers are often exposed to higher health risks, especially from infectious diseases. The GeoSentinel-Network, consisting of worldwide distributed travel clinics, identified important illnesses associated with international travels. Besides systemic febrile illnesses and dermatologic disorders, diarrheal illnesses are a main risk for international travelers [1]. This disease entity is described as travelers diarrhea (TD) with classic TD defined as the occurrence of three or more unformed stools in 24 hours in combination with at least one additional symptom like urgency, abdominal cramps, fever, or bloody diarrhea [2]. Although TD rates have declined as a result of improved hygienic conditions in the destination countries, TD still affects up to 30% of all travelers [3, 4]. The risk for TD depends on the type and destination of the travel. TD usually starts in the first week of the travel and is commonly a self-limiting disease lasting usually up to 5 days [5]. In about 75% of cases in which detection of the responsible pathogen was possible bacterial pathogens were the cause of TD, especially intestinal pathogenic* Escherichia coli*,* Salmonella* spp.,* Shigella* spp., and* Campylobacter jejuni* [6, 7]. Other important pathogens are viruses including norovirus and rotavirus affecting up to 15% of travelers with TD [6–8]. In the case of persistent diarrhea lasting more than 14 days protozoa like* Giardia lamblia* or* Entamoeba histolytica* have to be considered [7]. As most of the cases of TD are resolved before the end of the travel and sufficient laboratory diagnostic capacities are seldom available at the travel destinations, data on the etiologic spectrum of TD occurring during the periods of travel is scarce. As conventional stool samples are inconvenient to handle for the traveler and an adequate cooling chain is difficult to maintain in most travel destinations, the conduct of studies concerning the etiology of TD is hampered. Conventional Hemoccult stool cards (Beckman Coulter Inc., Brea, CA, US) are an accepted tool for the collection of stool samples in order to test for the existence of occult blood. They are easy to handle and can be hygienically stored during travel. Grimes et al. have shown that the detection of enteropathogens, especially enterotoxigenic* E. coli* and enteroaggregative* E. coli* from Hemoccult cards by polymerase chain reaction (PCR), can be successfully achieved for up to 14 months of storage at room temperature [9]. Commercially available multiplex PCR test-kits like the Gastrointestinal Pathogen Panel (GPP, Luminex, Austin, TX, USA) allow the simultaneous detection of up to 15 enteropathogens causing TD. Therefore, sampling on stool cards in combination with a multiplex PCR may be a convenient tool for testing stool samples for the etiology of TD.

To evaluate the performance of Hemoccult cards for stool sampling in combination with the GPP for the detection of model bacterial, viral, and protozoal TD pathogens, this study determined the LPDs and, if available, the LPCs were thereof calculated. LPDs and LPCs were then compared between conventional stool samples and Hemoccult cards. Subsequently, samples on stool cards were tested after a prolonged time interval simulating a travel duration of up to 6 weeks.

## 2. Material and Methods

### 2.1. Preparation of the Spiked Stools Samples and Norovirus GI/II Samples for Determining the LPDs/LPCs and for Storage on Hemoccult Cards

Six different formed human stool samples were obtained from our outpatient laboratory as matrices for spiking with* C. jejuni *or* E. histolytica*. These samples had been tested negative for enteropathogenic bacteria (*C. jejuni*,* Shigella* spp., and* Salmonella* spp.) by routine culture and for intestinal parasites by microscopic stool examination in the laboratories of the Department for Infectious Diseases and Tropical Medicine (DITM, accredited according to DIN EN ISO 15189). Samples were additionally tested negative for* E. histolytica* by PCR with the GPP. After routine testing, the samples were stored at −20°C until further processing in this study.

*C. jejuni subsp. jejuni *cultures (DSM Nr. 4688 Mar 09) were inoculated on blood agar from cryobeads and were cultivated at 42°C for 1-2 days. One loop-full (Hard Loop 10 *μ*L, VWR, Brescia, Italy) bacterial colonies were harvested and dissolved in 1 mL PBS.

*E. histolytica* samples (HK9 strain) stored in liquid nitrogen were thawed and grown in axenic culture in culture flasks using TYI-S33 culture medium at 37°C for 3-4 days, yielding only trophozoites [10]. Viable amoeba were mobilized from the inside wall by placing the culture flask into an ice water bath and were harvested according to standardized procedures as established in the accredited diagnostic laboratory of the DITM. Viable amoeba were dissolved in 1 mL PBS.

For determination of the concentration of* Campylobacter jejuni* and* E. histolytica*, 10 *μ*L of the pathogen solution was placed onto a Neubauer “improved” disposable counting chamber (C-Chip, Biochrom, Berlin, Germany) and counted according to the manufacturers recommendations. For counting* C. jejuni*, a cold Ziehl-Neelsen stain was done [11]. For* E. histolytica, *every visible trophozoite was counted without staining.

For* C. jejuni* 100.000 organisms/mL and for* E. histolytica* 5500 organisms/mL were defined as the intended concentration in the spiked stock stool sample to achieve a value well above the limits of detection (LoDs) of the GPP (*C. jejuni*: 60000 CFU/mL,* E. histolytica*: 2150 cells/mL) for these organisms as indicated by the manufacturer [12]. Usual pathogen loads for* C. jejuni* are 10^6^ to 10^9^ colony forming units (CFUs) per g stool [13]. Correct data on the pathogen load of* E. histolytica* in stool samples of patients are not readily available, as the evaluation by microscopy in stool is unreliable and cannot discern between pathogenic and apathogenic* Entamoeba* spp. [14]. Older literature data suggest that* Entamoeba* infection will be detected by microscopy in two stool examinations at a cyst excretion rate of 100.000 to 1.000.000 cysts per day [15]. Three grams of the negative stool matrices was diluted with PBS to a final volume of 30 mL, respectively, to achieve a diarrhea stool-like consistency and to ensure an adequate amount of material for testing of native samples and loading of the stool cards.

A part of each of the fluid stool matrices was then combined with the calculated amount of culture fluid to achieve the desired concentration of pathogens and a total volume of 5 mL of spiked stock stool sample for further testing.

Additionally, six frozen norovirus GI and six frozen norovirus GII stool samples of patients were obtained from a reference laboratory where they had been tested positive by a quantitative in-house PCR (the corresponding cross-threshold (Ct) values were provided by the reference laboratory; Tables 1 and 2). Pathogen load for the norovirus samples was unfortunately not available. The Ct values of the samples were well in the range of detection of the GPP. The corresponding LoDs of the GPP for norovirus GI and GII are Ct 40.85 and Ct 37.16, respectively [12]. After thawing, three grams of each norovirus-stool sample was diluted in phosphate buffered saline (PBS) to create a final sample volume of 5 mL with diarrhea-like consistency. For the storage trial a set of three norovirus GI and three norovirus GII stool samples and for the serial dilutions a different set of three norovirus GI and three norovirus GII stool samples were used.

### 2.2. Preparation of Serial Dilutions for* C. jejuni*,* E. histolytica*, and Norovirus GI/GII

For the serial dilutions, fluid stool matrices, pathogen culture fluids, and spiked stock stool samples for* C. jejuni* and* E. histolytica* were prepared as described above. For* C. jejuni* and* E. histolytica *the dilution steps 1 : 1 (pathogen content:* C. jejuni*: 100.000 organisms/mL,* E. histolytica*: 5500 organisms/mL), 1 : 2, 1 : 4, 1 : 8, and 1 : 16 were prepared. For creating each dilution step a decreasing amount of pathogen culture fluid was filled up with fluid stool matrices to achieve a final volume of 2 mL.

For three norovirus GI and GII samples, respectively, dilution series consisting of the original pathogen stool sample and the dilution steps 10^−2^, 10^−4^, 10^−6^, and 10^−8^ were prepared. To generate the dilutions 1 mL of the original pathogen stool sample dilution was added to 9 mL of PBS. To achieve the desired concentrations this procedure was repeated using 1 mL of the last dilution step after vigorous vortexing.

Besides the conventional samples of the dilution series, for each sample and each step of the dilution series one Hemoccult card was prepared. The two detection fields were loaded with 200 *μ*L of norovirus suspension or spiked stool suspension.

### 2.3. Stability during Storage: Loading of Hemoccult Cards

**Figure 1:**
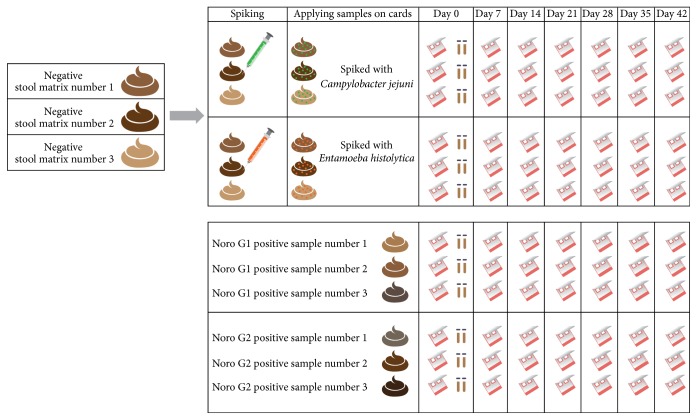
Schematic description of the preparation of the native and stool card samples for the storage trial.

For the storage trial, three samples of norovirus GI and norovirus GII, three* Campylobacter*-spiked stool matrices (pathogen content: 100.000 organisms/mL), and three* Entamoeba*-spiked stool matrices (pathogen content: 5500 organisms/mL) were used. For each of these samples seven Hemoccult cards were prepared for duplicate testing at time points 0, 7, 14, 21, 28, 35, and 42 days as described above (Figure 1). Furthermore, on day 0 for* C. jejuni* and* E. histolytica* samples of the unspiked stool solution, spiked stock stool sample, and the pathogen solution were prepared for testing. Concerning the norovirus samples only the diluted stool samples were additionally tested on day 0. The loaded Hemoccult cards were stored in sealed zipper bags (without desiccants or oxygen absorbers) at room temperature for the whole duration of the storage trial.

### 2.4. Extraction of Samples on Cards and Conventional Stool Samples by NucliSENS MiniMag

For the extraction of the card samples, the two detection fields of the Hemoccult cards were cut out using sterile single-use surgical blades and forceps and transferred into vials containing 2 mL NucliSENS lysis buffer (bioMérieux SA, Marcy l'Etoile, France). For extraction of the conventional stool samples, a sample volume of 200 *µ*l of the unspiked stool solutions, spiked stock stool samples, and the pathogen solutions was added to 2 mL lysis buffer. Samples were incubated for 40 minutes (200 rpm, room temperature) in a thermoshaker (Ditabis, Pforzheim, Germany) before centrifugation at 4000 rpm for five minutes. The supernatant was then used for further processing. The extraction procedure was done according to the manufacturers recommendation and the RNA/DNA extracts were resuspended in 50 *μ*L of elution buffer. DNA samples were immediately stored at −20°C and RNA samples at −80°C until further processing.

### 2.5. Detection of Gastrointestinal Pathogens with the GPP

10 *μ*L of extracted DNA/RNA samples was used for detection with the GPP according to the manufacturer's recommendation. In brief, a (reverse transcription) PCR was done. The PCR product was then combined with the detection beads included in the GPP and amplicons were detected by a MagPIX (Luminex, Austin, Texas, USA).

The LPDs were determined as the dilution step at which both duplicates of the respective stool card or native sample tested positive. The LPCs were, if available, then calculated from the LPDs.

### 2.6. Retesting of Samples with Discordant Results

Discordant PCR results between the tests of conventional and stool card samples were retested with the GPP. If discrepancies persisted, the samples were furthermore subjected to a commercial real-time PCR for detection of the relevant pathogen (Microgen, London, UK) according to the manufacturer's recommendation on a CFX96 real-time detection system (BioRad, Munich, Germany).

## 3. Results

### 3.1. Serial Dilutions of* C. jejuni* and* E. histolytica* Samples

The detection on stool cards was possible up to the dilution step of 1 : 8 mostly matching the detection in the conventional samples. In one* C. jejuni* containing sample the LPD of the conventional sample was one step lower (1 : 4) than the card sample (Table 1).

The* E. histolytica* card samples could be tested consistently positive up to the dilution step of 1 : 16. One conventional sample tested positive one dilution step lower (1 : 8) (Table 1).

### 3.2. Serial Dilutions of Norovirus GI/GII Samples

Concerning the norovirus samples, the LPDs for the norovirus GI and GII samples varied significantly between the different initial samples ranging from 10^−2^ to the dilution step of 10^−8^. The results showed a trend towards more sensitivity in stool card samples. One norovirus GI and two norovirus GII card samples could be tested positive one dilution step higher (10^−8^, 10^−8^, and 10^−4^, Table 1) than the corresponding conventional sample. On the other hand, in one norovirus G II sample the LPD was one step lower (10^−4^, Table 1) in the conventional sample than in the card sample.

### 3.3. Stability during Storage

On day 0 the pathogen solutions for* C. jejuni* and* E. histolytica* and the native stool samples for norovirus GI and GII were tested positive according to the expected pathogen. Testing of the stool card samples at the designated time points of days 0, 7, 14, 21, 28, 35, and 42 showed positive results for* C. jejuni* in 41/42 samples (detection rate of 97.6%) and all samples of* E. histolytica* tested positive (detection rate of 100%). Concerning the norovirus GI samples, 41/42 samples tested positive (detection rate of 97.6%) and all norovirus GII samples tested positive (detection rate of 100%) (Table 2). One norovirus GI sample tested consistently positive for* Cryptosporidium *spp. (Table 2). The unexpected positive result for* Cryptosporidium *spp. could be confirmed in the original stool sample by microscopy and antigen testing by ProSpecT Cryptosporidium Microplate Assay (Oxoid, Hants, UK).

## 4. Discussion

This study evaluated Hemoccult cards as a collection and storage tool of stool samples in combination with a commercially available multiplex PCR able to detect viral, bacterial, and protozoal organisms relevant for the etiology of TD. In the dilution trial the native samples and the samples on stool cards of* C. jejuni* and* E. histolytica* reached mostly consistent LPCs showing no reduced detection of the expected pathogens when stool cards were used as a sample collection tool. Comparing the native and stool card norovirus samples the results suggest that detection from stool cards is also similar to conventional stool samples. As we could perform only two replicates at each concentration to limit the costs for the testing, our results remain preliminary and larger studies are needed to confirm the LPDs/LPCs and the reproducibility. As the LPDs/LPCs concerning the conventional and card samples were all below the LoDs of the GPP, a loss of sensitivity using the Hemoccult cards is unlikely.

Lalani et al. used FTA Elute Cards for sample storage and found also comparable detectable concentrations for* C. jejuni* and norovirus in the conventional and card samples [16]. After one week of storage on FTA cards the detectable concentrations for* C. jejuni* on card samples were 10^5^ CFU/g and 10^3^ plaque forming units (PFU) for norovirus GI and norovirus GII. In the present study determination of CFUs for* C. jejuni* was not performed and the relationship between CFUs and bacterial count is therefore unclear. The LPCs were ranging from 25.000 bacteria/g (dilution step 1 : 4) to 12.500 bacteria/g (dilution step 1 : 8). In our study the provided norovirus samples showed a wide range of initial norovirus concentration. The LPDs for the different samples ranged from dilution step 10^−2^ to dilution step of 10^−8^ (Table 1). These results could be confirmed after repeating the testing with a commercially available real-time PCR for norovirus (Microgen). As the results were comparable between native and Hemoccult card samples, usage of Hemoccult cards is also feasible at varying norovirus concentrations.

In the storage trial the model pathogens (*C. jejuni*,* E. histolytica*, norovirus) could be detected reliably from the card samples at weekly intervals for up to six weeks. Grimes et al. could show the possibility of storage of* E. coli* stool samples on cards with successful detection by PCR for up to 14 months [9]. In contrast to this study no developer/fixative was used on the card samples of the present study. This increases the feasibility of the method as only a small amount of stool has to be put on the detection field and no additional developer/fixative fluid has to be carried along by the traveler. Additionally, in the context of an epidemiologic study no costs for this substance for each traveler will accrue. Lalani et al. could also successfully store stool samples spiked with several bacterial, protozoal, and viral pathogens including* C. jejuni* and norovirus GI/II for up to three months on FTA Elute Cards although* C. jejuni* was only detectable for up to one month [16]. Compared to Hemoccult cards FTA Elute Cards are more expensive and Hemoccult cards may be more convenient for travelers as the detection fields are readily covered by a carton wrapping and lid to prevent spoiling.

Several points warrant attention. Norovirus samples acquired from the reference laboratory were sent there for exclusive testing for norovirus from different unknown sources. Information on further pathogens in these samples was not available to us. Therefore, the detection of additional pathogens like the* Cryptosporidium *spp. in one norovirus GI sample can be explained. By chance, this showed that another protozoal pathogen could be stored and detected on stool cards for up to six weeks. This is in contrast to the results of Lalani et al. who had difficulties detecting* Cryptosporidium *spp. from spiked card samples [16]. Possibly, although we have only one sample with this pathogen, this could be an effect of our different extraction method using MiniMag. This result warrants further investigation.

As the study was done as a proof of concept study, storage was checked only at room temperature. Storage at higher temperature simulating travel to tropical countries was not checked, as the cost of GPP testing is very high and the number of samples and testing processes was therefore restricted. On the other hand, Lalani et al. found no effect of varying environmental conditions when the card samples were stored at 4°C or 31°C with the exception of* Cryptosporidium* spp. and* C. jejuni* that could be either poorly or not detected in that setting [16].

Additionally, in order to limit the cost of testing we could not repeat our examinations with unmodified infected stool samples and only a limited number of replicates could be performed. Unfortunately, the viral loads cannot be reported for the used norovirus samples and only Ct values can be shown semiquantitatively as these were samples from routine clinical testing and therefore not exactly quantified. On the other hand, the viral loads in our samples should thus reflect viral loads seen in norovirus patients in general.

Altogether, this study suggests that stool cards are a reliable tool for collecting stool samples for multiplex PCR-testing. Storage for up to 6 weeks at room temperature allows detection of viral, bacterial, and protozoal organisms. Therefore, this method could for example be used in studies on the etiology of TD or in the evaluation of prophylactic measures as the use of antibiotics or vaccines. Further studies on the feasibility of these cards during travel are therefore needed.

## Figures and Tables

**Table 1:** LPDs and LPCs of the serial dilutions for norovirus GI/GII, *C. jejuni*, and *E. histolytica* native and card samples.

Pathogen	Conventional sample			Stool card sample		
	Sample 1	Sample 2	Sample 3	Sample 1	Sample 2	Sample 3
Norovirus GI	10^−2^^a^	10^−2^^a^	10^−6^^a^	10^−2^^a^	10^−2^^a^	10^−8^^a^
Norovirus GII	10^−6^^a^	10^−4^^a^	10^−2^^a^	10^−8^^a^	10^−2^^a^	10^−4^^a^
*C. jejuni*	1 : 8	1 : 4	1 : 8	1 : 8	1 : 8	1 : 8
	12.500/mL	25.000/mL	12.500/mL	12.500/mL	12.500/mL	12.500/mL
*E. histolytica*	1 : 16	1 : 8	1 : 16	1 : 16	1 : 16	1 : 16
	344/mL	688/mL	344/mL	344/mL	344/mL	344/mL

LPCs are stated as calculated pathogen concentration (organisms/mL). ^a^Dilution steps retested by Microgen real-time PCR. Ct of norovirus (provided by reference laboratory): GI sample 1, Ct 20; GI sample 2, Ct 20; GI sample 3, Ct 18; GII sample 1, Ct 17; GII sample 2, Ct 18; GII sample 3, Ct 18.

**Table 2:** Results of the storage trial for *C. jejuni*, *E*. *histolytica*, and norovirus GI/II.

Sample^a^	Duplicate	Day 0	Day 7/14/21	Day 28	Day 35/42	Detection rates
*C. jejuni* Samples 1, 2	1	pos	pos	pos	pos	97.6%
	2	pos	pos	pos	pos	
*C. jejuni* Sample 3	1	pos	pos	**neg**	pos	
	2	pos	pos	pos	pos	

*E. histolytica *Samples 1, 2, 3	1	pos	pos	pos	pos	100%
	2	pos	pos	pos	pos	

Norovirus GI Sample 1	1	pos	pos	pos	pos	97.6%
		**Crypto**.	**Crypto.**	**Crypto.**	**Crypto.**	
	2	**neg**	pos	pos	pos	
			**Crypto.**	**Crypto.**	**Crypto.**	
Norovirus GI Samples 2, 3	1	pos	pos	pos	pos	
	2	pos	pos	pos	pos	

Norovirus GII Samples 1, 2, 3	1	pos	pos	pos	pos	100%
	2	pos	pos	pos	pos	

pos, positive; neg, negative; Crypto, *Cryptosporidium *spp.

^a^Pathogen content of samples: *C. jejuni*: 100.000 organisms/mL and *E. histolytica*: 5500 organisms/mL; Ct of norovirus (provided by reference laboratory): GI sample 1, Ct 20; GI sample 2, Ct 22; GI sample 3, Ct 23; GII sample 1, Ct 11; GII sample 2, Ct 16; GII sample 3, Ct 19.
