# Corrigendum to “Detection of Gastrointestinal Pathogens from Stool Samples on Hemoccult Cards by Multiplex PCR”

**DOI:** 10.1155/2017/7360509

**Published:** 2017-05-30

**Authors:** Martin Alberer, Nicklas Schlenker, Malkin Bauer, Kerstin Helfrich, Carolin Mengele, Thomas Löscher, Hans Dieter Nothdurft, Gisela Bretzel, Marcus Beissner

**Affiliations:** Department of Infectious Diseases and Tropical Medicine, Medical Centre, Ludwig-Maximilians-University (LMU), Munich, Germany

In the article titled “Detection of Gastrointestinal Pathogens from Stool Samples on Hemoccult Cards by Multiplex PCR” [1], there was an error in Acknowledgments, which should be corrected as follows.

“The authors thank Dr. Florian Battke, Dr. Battke SCIENTIA Inc., for his scientific support. This paper is the result of the doctoral thesis of Nicklas Schlenker. The project was supported by research grants from the Friedrich Baur Foundation, Takeda Inc., and Luminex Inc.”
